# Divergent effects of norepinephrine, dopamine and substance P on the activation, differentiation and effector functions of human cytotoxic T lymphocytes

**DOI:** 10.1186/1471-2172-10-62

**Published:** 2009-12-08

**Authors:** Carina Strell, Anne Sievers, Philipp Bastian, Kerstin Lang, Bernd Niggemann, Kurt S Zänker, Frank Entschladen

**Affiliations:** 1 Institute of Immunology, Witten/Herdecke University, 58448 Witten, Germany

## Abstract

**Background:**

Neurotransmitters are important regulators of the immune system, with very distinct and varying effects on different leukocyte subsets. So far little is known about the impact of signals mediated by neurotransmitters on the function of CD8^+ ^T lymphocytes. Therefore, we investigated the influence of norepinephrine, dopamine and substance P on the key tasks of CD8^+ ^T lymphocytes: activation, migration, extravasation and cytotoxicity.

**Results:**

The activation of naïve CD8^+ ^T lymphocytes by CD3/CD28 cross-linking was inhibited by norepinephrine and dopamine, which was caused by a downregulation of interleukin (IL)-2 expression *via *Erk1/2 and NF-κB inhibition. Furthermore, all of the investigated neurotransmitters increased the spontaneous migratory activity of naïve CD8^+ ^T lymphocytes with dopamine being the strongest inducer. In contrast, activated CD8^+ ^T lymphocytes showed a reduced migratory activity in the presence of norepinephrine and substance P. With regard to extravasation we found norepinephrine to induce adhesion of activated CD8^+ ^T cells: norepinephrine increased the interleukin-8 release from endothelium, which in turn had effect on the activated CXCR1^+ ^CD8^+ ^T cells. At last, release of cytotoxic granules from activated cells in response to CD3 cross-linking was not influenced by any of the investigated neurotransmitters, as we have analyzed by measuring the β-hexosamidase release.

**Conclusion:**

Neurotransmitters are specific modulators of CD8^+ ^T lymphocytes not by inducing any new functions, but by fine-tuning their key tasks. The effect can be either stimulatory or suppressive depending on the activation status of the cells.

## Background

Almost two decades ago the observation has been made that lymphoid organs are directly innervated, mostly by neuropeptidergic fibers, and the question was raised whether the supplied neurotransmitters might have immunomodulatory functions [1,2]. This finding provided an anatomical rationale for the investigation of the effects of neurotransmitters on leukocytes, especially on B and T lymphocytes. It turned out, that several neurotransmitters have very distinct and varying functions on different leukocyte subsets (for overview see [3]). However, up to now there is no clear pattern of how the neuro-endocrine system in its function as the superordinate regulatory organ of the body modulates the immune system in common. This is due to the complexity of both organ systems and their multilayer interaction. Consequently, the discussion is still ongoing if and how emotions and sensations are translated into a general stimulation or suppression of the immune system.

Nevertheless, a large number of reports have been published that describe the function of neurotransmitters on certain leukocytes. Best characterized is probably the function of norepinephrine. This neurotransmitter is of special interest, since it is not only locally released from sympathetic nerve cells, but is also systemically disseminated after release from the adrenal gland. Furthermore, catecholaminergic innervation of lymph nodes increases under psycho-social stress conditions, as was shown on macaques [4]. T and B lymphocytes both express the β2-adrenoceptor, which is responsible for the intracellular signal transduction of norepinephrine. However, it is unclear whether both activated T helper (Th)1 and Th2 lymphocytes, or only activated Th1 lymphocytes express the β2-adrenoceptor [5]. In Th1 lymphocytes, norepinephrine has influence on the expression of interferon (IFN)-γ, depending on the time-point of its presence during activation: when norepinephrine was added before activation, IFN-γ production decreased; when added after activation, IFN-γ production increased [5]. The importance of this neuro-immunologic axis becomes even more obvious in patients with spinal cord injury, which have an impaired response to infections. In a mouse model it has been shown that, depending on the level of spinal cord injury, increased concentrations of circulating corticosterone and norepinephrine are present, which lead to an impaired antibody synthesis [6]. However, β2-adrenergic stimulation or cyclic adenosine-monophosphate (cAMP) accumulation - which is a key signalling event caused by this receptor - elicit in concert with other stimuli divergent effects in B cell subsets concerning proliferation, B7-2 and major histocompatibility complex II expression, differentiation to antibody-secreting cells, and antibody production [7]. Interestingly, the antibody production largely depends on the duration of cAMP accumulation. Short term elevation of the cellular cAMP concentration results in an increase of antibody production, whereas a long term elevation decreases antibody production [7]. A recent work by Grebe et al. reported that β-blockers such as nadolol enhance antiviral CD8^+ ^T lymphocyte responses in mice, suggesting an immuno-suppressive effect of norepinephrine [8].

Dopamine is the metabolic precursor of norepinephrine. The secondary lymphoid tissues are abundantly innervated by the sympathetic nerves that store a large amount of dopamine [9]. The D1 to D5 receptors for dopamine are differentially expressed on leukocyte subsets [10-13], and it has also been reported that dopamine can act via β-adrenoceptors [14]. Dopamine suppresses the proliferation and cytotoxicity of T lymphocytes [15], but stimulates adhesion to fibronectin [11]. With regard to the cytokine release, the function of dopamine is not clear. Depending on the experimental setting, dopamine has been reported to have increasing [16] as well as decreasing [17] function. In the first study, the peripheral blood mononuclear cell (PBMC) fraction was activated with anti-CD3 and anti-CD28 antibodies [16]. In the second study, the T lymphocytes were further purified from the PBMC fraction and activated only with an anti-CD3 antibody [17].

Substance P is a peptide of the neurokinin family, which plays a role in depressive disorders [18], and inflammatory processes [19,20]. Substance P is also released by peripheral nerve endings that innervate lymphoid organs [21]. In contrast to dopamine, substance P inhibits T lymphocyte adhesion to fibronectin [22], and in contrast to norepinephrine, substance P increases the number of immunglobulin-secreting cells [23].

Unfortunately, there exists no general scheme to predict how neurotransmitter will act on leukocytes, in contrast the effect of a neurotransmitter depends not only on the parameter of interest but also on the cell type investigated and its activation state or phenotype as well as on the lymphoid tissue from which the cells derive [22].

This brief introduction shows that norepinephrine, dopamine and substance P have diverse, sometimes opposite effects on B and Th lymphocytes. In comparison to these lymphocyte subpopulations, the influence of neurotransmitters on the function of CD8^+ ^T lymphocytes is much less investigated. Therefore, we conducted the present study in order to complement the picture of neurotransmitter action on leukocyte subsets. The CD8^+ ^T cells have to be activated in the lymph node by their T cell receptor. They then emigrate from the lymph node, extravasate from the blood stream at infected or inflamed tissue sites and migrate through the tissue in search for target cells. After recognition of these cells, they eliminate these cells. We investigated the influence of norepinephrine, dopamine and substance P on these key functions of CD8^+ ^T lymphocytes *in vitro*: activation, migration, extravasation and cytotoxicity. Beyond this, we provide molecular explanations on how the neurotransmitters affect these cells functions.

## Results and Discussion

### Impact of neurotransmitters on the activation of CD8^+ ^T lymphocytes depends on IL-2

**Figure 1:**
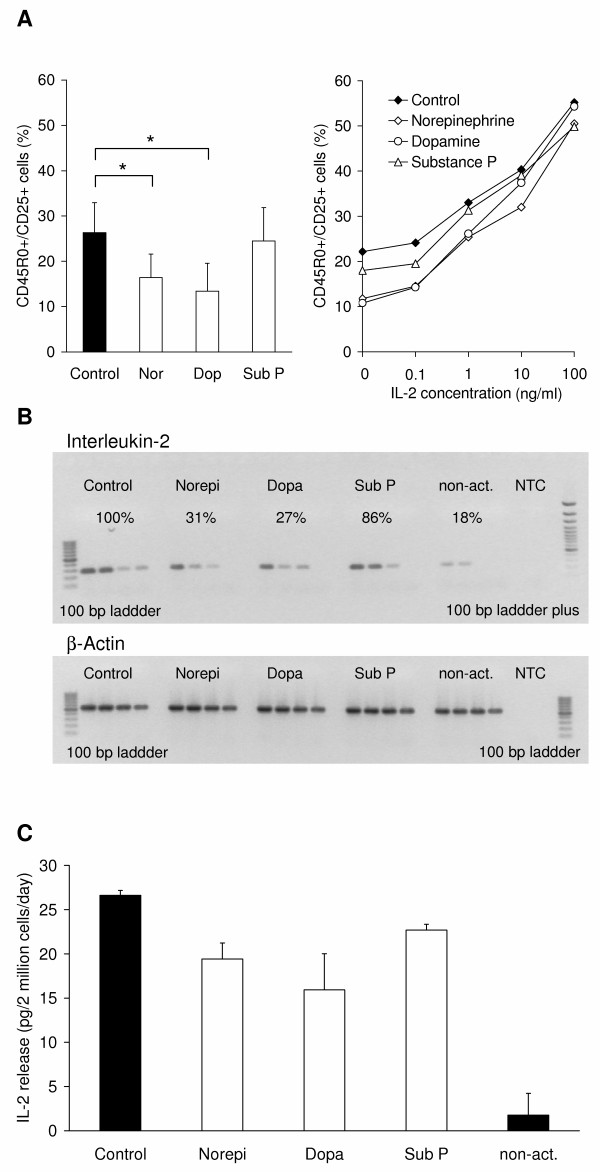
**Activation of CD8^+ ^T lymphocytes with anti-CD3 and anti-CD28 antibodies**. The activation status was detected by double-staining for the activation markers CD25 and CD45R0. (A) Flow-cytometrical analysis of CD8^+ ^T lymphocytes activated in the presence of neurotransmitters (10 μM norepinephrine, 1 μM substance P and dopamine). The graph shows the mean values and standard deviation of double-positive T lymphocytes of four independent experiments with cells from different donors. Asterisks mark statistically significant changes (p < 0.05). Stepwise addition of external IL-2 reverses the inhibitory effect of the neurotransmitters, but in turn leads to a higher number of CD25^+ ^CD45RO^+ ^lymphocytes. (B) Expression of IL-2 RNA during activation under the influence of neurotransmitters. RT-PCR was performed with cDNA in the dilution steps 1:125 1:625 1:3125 and 1: 15625. Per lane, 10 μl of PCR sample were applied to the agarose gel. Beta-actin expression was used as house-keeping gene. (C) The release of IL-2 by CD8^+ ^lympocytes during activation in the presence of neurotransmitters was measured by using an enzyme-linked immunoassay. The graph shows mean values and standard deviation of three independent experiments with cells from different donors.

CD8^+ ^T lymphocytes were activated by cross-linking of the T cell receptor molecules CD3 and CD28 by immobilized antibodies. Activation was monitored by staining of the classical activation markers CD45R0 and CD25. After four days of activation 26.4 ± 6.6% of the CD8^+ ^T lymphocytes were double positive for CD45R0 and CD25 (Fig. 1A; left). Norepinephrine and dopamine inhibited the activation to 16.4 ± 5.2% and 13.4 ± 6.2% of the cells, respectively, whereas both effects were significant (p = 0.040 and p = 0.026). Substance P led only to a slight, non-significant reduction (25.5 ± 7.3% double-positive cells). This inhibition of activation was not due to cell death, as we have analyzed by flow-cytometry. IL-2 is a well known cytokine in T cell activation, and consequently the exogenous addition of IL-2 strongly supports the activation process. The stepwise addition of IL-2 (0.1, 1, 10 and 100 ng/ml) had two effects: firstly, the more IL-2 was added, the weaker the inhibitory effects of the neurotransmitters were. Secondly, the number of CD45R0 and CD25 double-positive T lymphocytes increased dose-dependently (Fig. 1A; right). None of the investigated neurotransmitters norepinephrine, dopamine or substance P had a significant influence on the activation in the presence of 100 ng/ml IL-2 (Fig 1A). Thus, we asked, whether the neurotransmitters might have an effect on the IL-2 expression. We analyzed the amount of IL-2 mRNA by semi-quantitative RT-PCR after 24 hours of activation (Fig. 1B). Cells treated with norepinephrine (10 μM) and dopamine (1 μM) showed a decrease in their level of IL-2 mRNA to 31% and 27%, respectively, when compared to control cells and adjusted to β-actin expression. Substance P treatment (1 μM) led to a moderate reduction to 86%, whereas non-activated cells showed only 18% expression of IL-2 RNA when compared to their activated counterparts (Fig. 1B). The RT-PCR results were further verified by IL-2 ELISA experiments to investigate if protein expression and secretion are also decreased. Therefore the cell culture supernatant after two days of activation was collected. Under control conditions CD8^+ ^T lymphocytes secreted 26.62 ± 0.58 pg IL-2 per 2 × 10^6 ^cells per day (Fig. 1C). In the presence of neurotransmitters the amount of secreted IL-2 was decreased (norepinephrine 19.43 ± 1.8, dopamine 15.94 ± 4.09, substance P 22.68 ± 0.67 pg IL-2 per 2 × 10^6 ^cells per day). Unactivated lymphocytes secreted only a small amount of IL-2.

These results deliver an explanation for the reduction of activation by the neurotransmitters, and it explains, why no effects of the neurotransmitters were observed, when IL-2 was added exogenously during activation. Norepinephrine and dopamine inhibit the IL-2 expression thus interfering with the generation of an autocrine IL-2 loop necessary for optimal activation of T cells.

**Figure 2:**
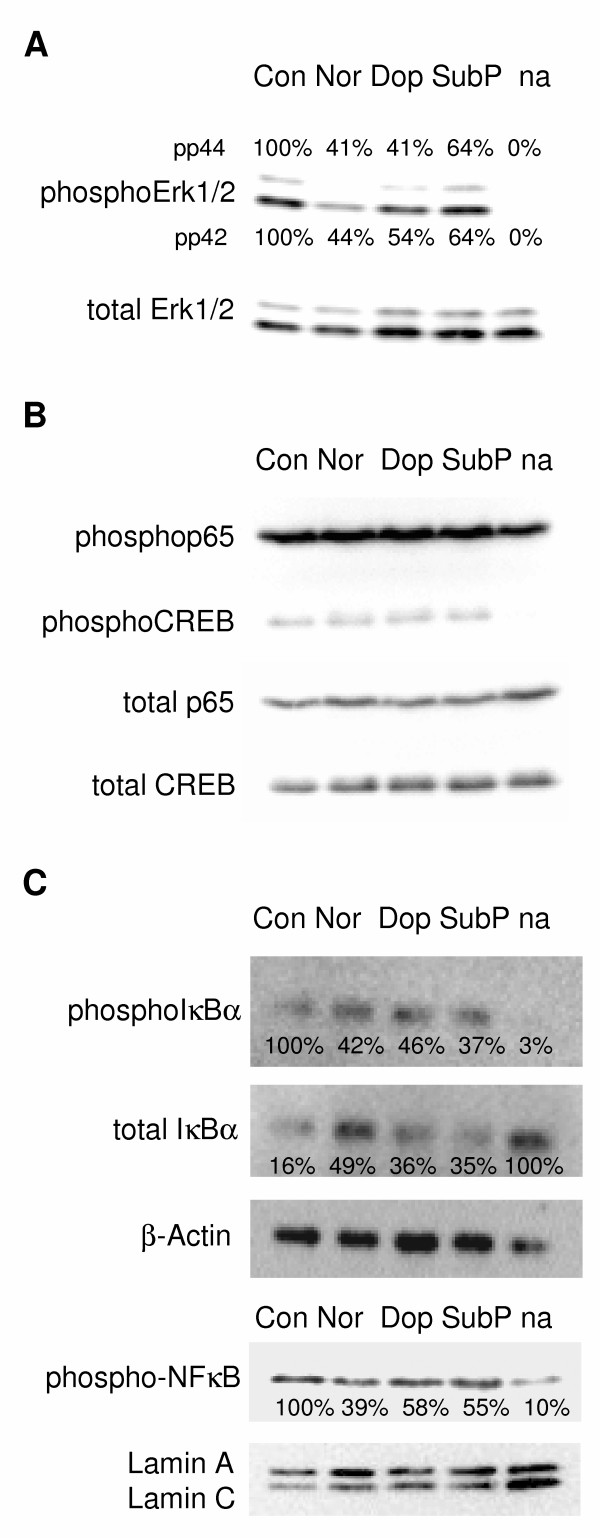
**Transcription factor activation**. (A) Immunoblots of total Erk1/2 and phosphorylated Erk1/2, (B) immunoblots of total and phosphorylated p65 and CREB, and (C) immunoblots of total and phosphorylated IκBα in CTL during activation, and of phospho-NF-κB in the nuclear fraction. β-Actin and Lamin A/C concentrations were used as standards to adjust the applied protein amounts. Abbreviations are: Con = control; Nor = norepinephrine; Dop = dopamine; SubP = substance P; na = non-activated control. (D) Neurotransmitters were added at 10 μM (norepinephrine) and 1 μM (substance P and dopamine) in all experiments.

The IL-2 promoter is under the control of several transcription factors. We thus investigated, whether the neurotransmitters might inhibit one or more of these transcription factors. Activator protein (AP)-1, nuclear factor of activated T-cells (NFAT), nuclear factor (NF)-κB, and the cAMP response element binding protein (CREB) are known to be necessary for optimal IL-2 gene transcription [24]. Phosphorylated Erk1/2 leads to an up-regulation of c-Fos expression [24,25] and c-Fos in turn, when dimerized with the Jun family of transcription factors, forms AP-1 [26]. Therefore, we investigated Erk1/2 phosphorylation as a marker for AP-1 activity (Fig. 2A). Erk1/2 phosphorylation was strongly reduced by norepinephrine (p44 to 41% and p42 to 44%) and dopamine (p44 to 41% and p42 to 54%) and to a minor degree by substance P (p44 and p42 to 64%; Fig. 2A). In the non-activated control, no Erk1/2 phosphorylation was detectable.

We next investigated the phosphorylation of the transcription factors p65 (NF-κB family) and CREB one hour after activation. Both of these transcription factors were highly phosphorylated in activated cells, and no differences in the induction of phosphorylation were observed with any of the used neurotransmitters (Fig. 2B). NF-κB is under the control of IκB, which restricts NF-κB to the cytoplasm and inhibits its DNA binding activity [27]. Upon activation of the CTL, IκB is phosphorylated and subsequently degraded (Fig. 2C; upper part), thereby releasing NF-κB and allowing its transition to the nucleus (Fig. 2C; lower part). When adjusted to the whole amount of IκB, norepinephrine reduced the phosphorylation to 42%, dopamine to 46%, and substance P to 37% (whereas in the non-activated cells 3% phosphorylation was measured). Due to this, a reduced degradation was detected in activated CD8^+ ^T lymphocytes. Compared to non-activated cells, only few IκB (16%) was detected in activated cells. Under neurotransmitter treatment, the remaining amount of IκB increased to 49% (norepinephrine), 36% (dopamine), and 35% (substance P). In consequence, a reduced amount of phosphorylated NF-κB was found in the nuclear fraction of the lymphocytes when treated with the neurotransmitters during activation: 39% with norepinephrine, 58% with dopamine, and 55% with substance P (Fig. 2C; lower part).

Transcription factors of the NFAT family are activated by calcium signalling via calmodulin/calcineurin. An involvement of NFAT in the inhibition of IL-2 expression is unlikely, since none of the neurotransmitters had a significant influence on the intracellular calcium increase in response to CD3/CD28 cross-linking (changes of the cytosolic calcium concentration were measured by flow cytometrical analysis on the basis of a protocol described by Gergely *et al*. [28]; data not shown).

T cell activation via CD3 and CD28 leads to activation of associated kinases of the src-family and the recruitment and activation of the tyrosine kinase ZAP-70 (ζ-chain associated protein). With regard to IL-2 transcription two key signalling pathways become activated. (1) phospholipase C gamma (PLCγ) is recruited to the membrane site and becomes activated. PLCγ cleaves phosphatidylinositol bisphosphate (PIP_2_) to yield inositol triphosphate (IP_3_) and diacylglycerol (DAG). DAG activates NFκB via protein kinase θ and IP_3 _opens intracellular calcium storages. The increased level of intracellular Ca^2+ ^activates the serine phosphatase calcineurin, which dephosphorylates NFAT. Dephosphorylated NFAT enters the nucleus. (2) CD3 and CD28 signalling leads to the activation of Ras. Ras induces the activation of the transcription factor AP-1 via the MAP kinase cascade Raf-1/MEK/ERK and further mediates the degradation of IκB via AKT.

In summary we could show that those transcription factors, which are under the control of Ras, *i.e. *AP-1 and IκB, are regulated by neurotransmitters [29,30], whereas those, which are regulated by downstream signals of the PLCγ, i.e. NF-κB and NFAT, are not affected [31,32].

### Effect of neurotransmitters on the migratory behaviour

**Figure 3:**
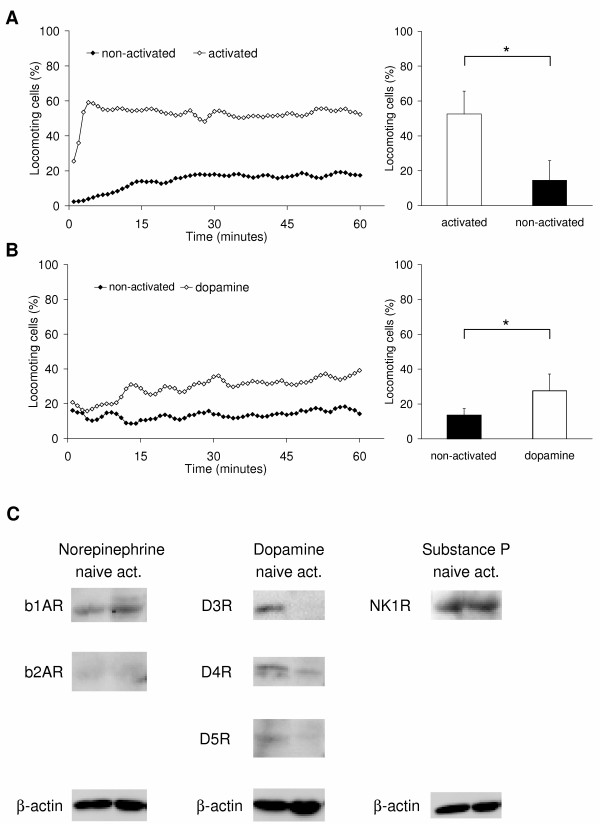
**Migratory activity of CD8^+ ^T lymphocytes**. Each graph shows on the left side the time-course of the mean migratory activity and on the right side the time-average of the migratory activity as mean value and standard deviation of the same experiments. In (A), nine experiments were performed with activated and naive CD8^+ ^T lymphocytes, in (B) four experiments were performed with naïve CD8^+ ^T lymphocytes (1 μM dopamine). For each experiment, blood from different donors was used. Asterisks mark statistically significant changes (p < 0.05). (C) Shows the western blot analysis of the neurotransmitter receptors expressed on naïve and activated CD8^+ ^T lymphocytes. β-Actin served as loading control.

**Figure 4:**
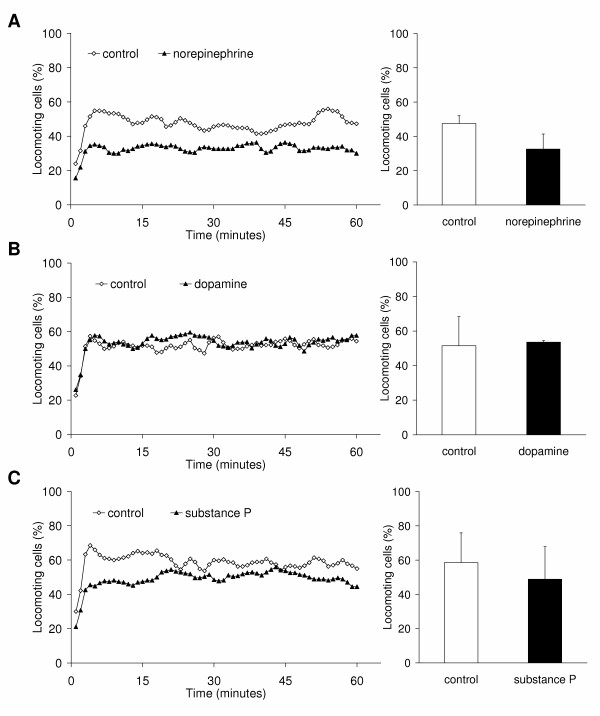
**Migratory activity of activated CD8^+ ^T lymphocytes**. Each graph shows on the left side the time-course of the mean migratory activity and on the right side the time-average of the migratory activity as mean value and standard deviation of the same experiments. Three experiments were performed with each neurotransmitter: (A) 10 μM norepinephrine, (B) 1 μM dopamine, (C) 1 μM substance P. For each experiment, blood from different donors was used. Asterisks mark statistically significant changes (p < 0.05).

Activated CD8^+ ^T lymphocytes showed a striking and significant (p < 0.001) increase of the migratory activity from 14.5 ± 11.4% to 52.5 ± 13.2% locomoting cells after activation (Fig. 3A). Furthermore, we have reported previously, that the migratory activity of naïve, non-activated CD8^+ ^T cells is increased by the treatment with norepinephrine or substance P [33]. Likewise, dopamine significantly (p = 0.034) increased the migratory activity from 13.6 ± 3.8% to 27.6 ± 9.6% locomoting cells (Fig. 3B). In contrast to these results, the migratory activity of activated T lymphocytes was reduced by norepinephrine and substance P, whereas dopamine had no effect (Fig. 4A to 4C). Norepinephrine almost significantly (p = 0.061) reduced the migration of activated CD8^+ ^T cells from 47.5 ± 4.6% to 32.6 ± 8.9% locomoting cells (Fig. 4A). Dopamine had no effect on the migratory activity (51.5 ± 16.8% locomoting cells control vs. 53.6 ± 0.9% locomoting cells; Fig. 4B). Substance P reduced the migration from 58.6 ± 17.4 to 48.9 ± 18.9% locomoting cells (Fig. 4C); this effect was however not significant due to the high standard deviation. These differences might be due to the fact, that dopamine receptors are down-regulated on activated CD8^+ ^T lymphocytes as we found by western blot analysis, whereas the β1-adrenoceptor and the NK-1 receptor are still expressed by activated cells (Fig. 3C). We found only the D3 and D4 receptors and to a lesser degree the D5 receptor to be expressed on naïve CD8^+ ^T lymphocytes. All three of them become down-regulated during T cell activation and with the exception of the D5 receptor these findings are in accordance with Watanabe et al. [12].

Thus, naïve and activated CD8^+ ^T lymphocytes differ in their level of spontaneous migratory activity. In the naïve state the spontaneous migratory activity is only moderate whereas in the activated state more than half of the cells show migratory activity. This observation is not surprising since the expression pattern of matrix adhesion molecules changes during the activation process and thereby the intracellular signalling. Consequently, neurotransmitters have distinct effects on naïve and activated CD8^+ ^T lymphocytes. Collagen I receptors such as very late antigen (VLA)-1 and VLA-2 are almost absent on naïve CD8^+ ^T cells but become upregulated during activation [34,35]. This might explain our observation that the naïve lymphocytes show less spontaneous migratory activity than the activated lymphocytes in our migration assay containing 95-98% collagen I as almost single matrix component, with the remainder being comprised of type III collagen.

Naïve CD8^+ ^T lymphocytes show an increase in their migratory activity in response to dopamine (Fig. 3B), norepinephrine and substance P [33], with dopamine being the strongest inducer. Our finding on dopamine is consistent with Watanabe *et al. *[12], who described that dopamine induces chemotaxis in CD45RA^+ ^naïve CD8^+ ^cells via D3 receptor and postulated a role for endogenous dopamine in the homing of naïve CD8^+ ^T cells.

### Adhesion of CTL to endothelium is selectively regulated by neurotransmitters

**Figure 5:**
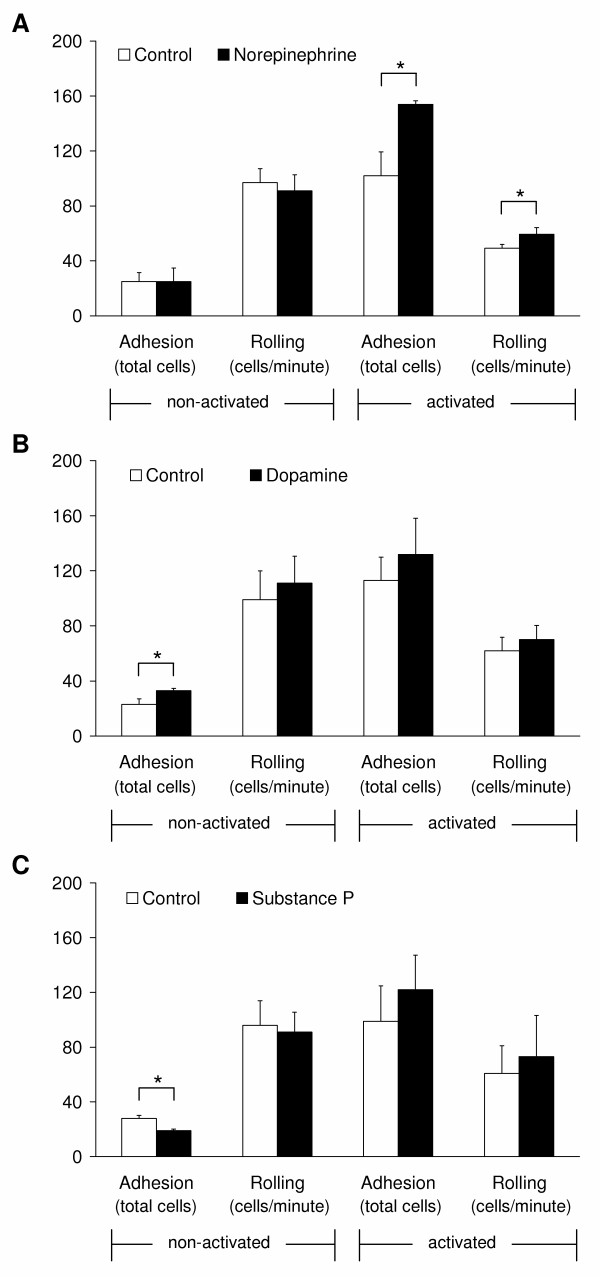
**Interaction of CD8^+ ^T lymphocytes with endothelial cells in a flow-through extravasation assay**. The left columns of each graph show the adhesion and rolling of non-activated cells, the right columns show the adhesion and rolling of activated cells. "Adhesion" is the total number of tightly adherent cells in a 20 minute observation period. "Rolling" is the number of cells per minute, which rolled on the endothelium before they either tightly adhered or were washed away. Graphs show mean values and standard deviation of three independent experiments for each neurotransmitter: (A) 10 μM norepinephrine, (B) 1 μM dopamine, and (C) 1 μM substance P. Asterisks mark statistically significant changes (p < 0.05).

The ability to extravasate is an important property of immune cells independent of their activation state. Naïve lymphocytes need to extravasate during the homing process and activated cells extravasate in order to reach tissue sites of inflammation or injury. The idea that neurotransmitters influence the extravasation process seems likely, because nerve fibres are particularly concentrated around vascular endothelial cells [9]. Non-activated CD8^+ ^T lymphocytes show only low adhesion to the endothelium, but a high rolling activity, as we have investigated by our flow-through adhesion assay (Fig. 5). In activated CD8^+ ^T lymphocytes, the adhesion significantly (p < 0.001) increased from 25.3 ± 2.5 to 104.7 ± 7.4 cells (average values of Fig. 5A to 5C). Due to the increased adhesion, the rolling activity is reduced. Both adhesion and rolling of activated CD8^+ ^cells is increased by addition of each of the investigated neurotransmitters directly to the experiment. Norepinephrine significantly (p = 0.007) increased the adhesion of the activated cells from 102 ± 17 to 154 ± 3 cells (Fig. 5A). Likewise, the rolling was significantly (p = 0.032) increased from 49 ± 3 to 59 ± 5 cells/min (Fig. 5A). Dopamine and substance P enhanced the adhesion and rolling of the activated cells only marginally, which was not statistically significant (Fig. 5B and 5C). With regard to naïve, non-activated CD8^+ ^cells, all of the investigated neurotransmitters had only minor effects. However, dopamine significantly (p = 0.014) increased the adhesion of the naïve, non-activated cells (Fig. 5B), whereas substance P led to a significant reduction (p = 0.004; Fig. 5C). With regard to dopamine, the finding is in accordance with results from other groups who described an increase in the adhesion of naïve CD45RA^+ ^CD8^+ ^lymphocytes to fibronectin and ICAM-1 in response to dopamine but not substance P [12,22]. This effect was exclusively mediated by the D3 receptor and led to an activation of the fibronectin receptors VLA-4 and VLA-5 [22], and the intercellular adhesion molecule (ICAM)-1 receptor lymphocyte function-associated antigen (LFA)-1 [12] on naïve lymphocytes. Thus dopamine seems to exhibit a unique function leading to the activation of integrin molecules in naïve CD8^+ ^cells which cannot be induced by norepinephrine or substance P. This observation supports the theory of dopamine being a specific homing factor of naïve CD45RA^+ ^CD8^+ ^cells [12].

**Figure 6:**
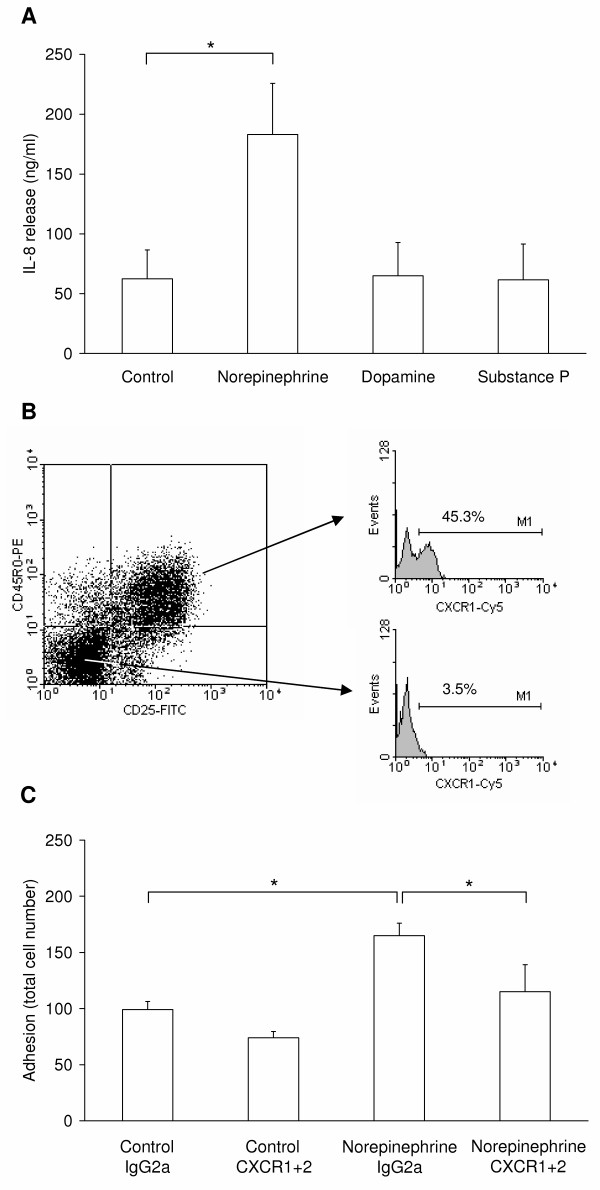
**IL-8 in the interaction of CD8^+ ^T lymphocytes and endothelium**. (A) The release of IL-8 from the endothelium in response to the investigated neurotransmitters (10 μM norepinephrine, 1 μM substance P and dopamine) was measured using an enzyme-linked immunoassay. The graph shows mean values and standard deviation of four independent experiments with cells from different donors. Asterisks mark statistically significant changes (p < 0.05). (B) Flow-cytometrical measurement of the CXCR1 expression on activated and naïve CD8^+ ^T lymphocytes. The cells were distinguished for their activation state by CD25 and CD45R0 expression (left). Each subpopulation was characterized for CXCR1 expression separately (right). (C) Extravasation experiments were performed with a blockade of the IL-8 receptors on the activated CD8^+ ^T lymphocytes by receptor-specific antibodies (abCXCR1+2). Isotypic antibodies (IgG2a) were used as control. Graphs show mean values and standard deviation of four independent experiments with cells from different donors. Asterisks mark statistically significant changes (p < 0.05).

Since the neurotransmitters were added directly to the cell suspension at the beginning of each experiment both cell types involved - the lympocytes as well as the endothelial cells- are exposed to the substance. Interestingly, norepinephrine, but not dopamine or substance P induced a significant (p = 0.013) increase of the IL-8 release from the endothelial cells (from 62.4 ± 24.1 to 182.9 ± 42.8 ng/ml; Fig. 6A), even when pre-activated with the cytokine IL-1β (since already IL-1β treatment alone leads to an increase of IL-8 release). Chemokines such as IL-8 are cationic proteins, which bind to heparansulfate and related glycosaminoglycan moieties on the endothelial monolayer [36,37], and thereby becomes presented to rolling cells. Several studies have conclusively shown that the recruitment of circulating leukocytes at vascular sites in target tissue is linked to activation of G_i_-protein signalling in leukocytes induced by chemokines presented on the apical site of the endothelial monolayer (reviewed in [38]). These immobilized chemokines are involved in the rapid modulation of the avidity of integrins such as VLA-4 [39] or LFA-1 expressed by lymphocytes. To confirm this hypothesis, we investigated the surface expression of the according IL-8 receptors CXCR1 and CXCR2 on CD8^+ ^lymphocytes: CXCR1 was almost absent on naïve cells (3.5% CXCR1^+ ^cells), but it became upregulated during activation (45.3% CXCR1^+ ^cells; Fig. 6B). We did not detect an expression of the IL-8 receptor CXCR2 neither on naïve nor on activated CD8^+ ^T lymphocytes (data not shown), which corresponds to the results of Takata *et al*. [40] and Nishimura et al. [41], but is in conflict with other findings [42]. To investigate a possible role for CXCR1 in the adhesion of CD8^+ ^T lymphocytes to endothelium in the presence of norepinephrine we blocked this receptor, and CXCR2 as well (because of the conflicting reports that the CXCR2 is expressed on activated CD8^+ ^cells [42]), *via *a specific blocking antibody (Fig. 6C). This blockade led to a significant (p = 0.022) reduction of the activated CD8^+ ^T lymphocyte adhesion to the endothelial cells in the presence of norepinephrine compared to the isotype (IgG2a) control from 165 ± 11 to 115 ± 24 adherent cells. This reduction was however not completely down to the control level (99 ± 7 and 74 ± 6 adherent cells with IgG2a and CXCR1/2 blocking antibodies, respectively; Fig. 6C). Thus, the effect of norepinephrine on the adhesion of activated CD8^+ ^T lymphocytes shown in Fig. 5A is not only mediated by IL-8. Instead the effect can be further mediated by other chemokines released by the endothelium or by a direct effect of norepinephrine on the T cells. Since we have just screened a subset of chemokines released by endothelial cells, we cannot exclude that the release of further chemokines is affected by norepinephrine.

From our experiments, we can exclude that substance P and dopamine as well act in the way as norepinephrine does, since these neurotransmitters do not result in an increased release of IL-8 from the endothelium.

### Degranulation is not influenced by neurotransmitters

**Figure 7:**
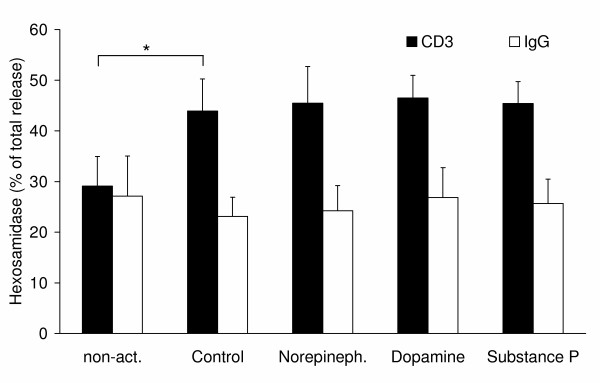
**Cytotoxicity of CD8^+ ^T lymphocytes as measured by β-hexosamidase release**. CD8^+ ^T lymphocytes were stimulated to release cytotoxic granules by CD3 cross-linking (black columns). A non-cross-linking isotypic IgG2a antibody was used as control (IgG; white columns). The graph shows mean values and standard deviation of five independent experiments with cells from different donors. Asterisks mark statistically significant changes (p < 0.05).

The effects of the neurotransmitters on the ability of the cells to degranulate were measured by β hexosamidase release (Fig. 7) in response to CD3 cross-linking, which mimics the recognition of a target cells without CD28 co-signalling. This process results in a calcium-dependent release of cytotoxic enzymes from intracellular granules that have been formed during activation. In naïve CD8^+ ^T lymphocytes, the CD3-induced release of β hexosamidase is as high as the release induced by an isotypic control antibody (Fig. 7; left). After activation, the CD3-induced release of β hexosamidase is significantly (p = 0.017) increased from 29.1 ± 5.8% to 43.9 ± 6.3% release. However, none of the investigated neurotransmitters caused any effect in activated CD8^+ ^T lymphocytes (Fig. 7). Determination of the calcium-signalling in response to CD3 cross-linking showed no differences, too (data not shown). In conclusion, it seems that the investigated neurotransmitters can affect the development of armed effector cells, but when these cells once have been formed, they cannot be inhibited in their effectory function by neurotransmitters.

## Conclusion

Although we have large and detailed knowledge how certain neurotransmitters act on particular leukocyte subpopulations, the remaining question is still how emotions and sensations are translated into modulatory effects of the immune system, and whether this at all plays a role in the function of the immune system [43].

Taken together, we have addressed the question how the neurotransmitters norepinephrine, dopamine and substance P influence the function of CD8^+ ^T lymphocytes. We have identified a regulation pattern (Table 1) showing that neurotransmitters can act either immunostimulatory or immunosuppressive. This depends mainly on the activation state of the T cells. We were able to assign downstream targets of the observed effects. Neurotransmitters cannot *per se *induce new functions in CD8^+ ^T lymphocytes, *i.e. *migration, adhesion and cytotoxicity, but they play an important role in the fine-tuning of T cell responses.

**Table 1:** Summary of neurotransmitter effects on CD8^+ ^T lymphocytes

		Norepi	Dopamine	Sub P
**Activation**		↓↓	↓↓	--
**Migration**	naive	↑	↑↑↑	↑
	activated	↓↓	--	↓
**Adhesion to**	naive	--	↑↑↑	↓↓
**endothelium**	activated	↑↑↑	↑	↑
**Degranulation**	activated	--	--	--

## Methods

### Cell isolation and activation

CD8^+ ^cells were isolated from heparinised human peripheral blood by a two step procedure. First, the lymphocyte-containing PBMC fraction was isolated by a density gradient centrifugation on lymphocyte separation medium (LSM 1077; PAA, Pasching, Austria). Subsequently, CD8^+ ^T lymphocytes were positively selected from the mononuclear fraction by immunomagnetic beads, which were coated with mouse anti-human CD8 monoclonal antibodies (Dynabeads; Invitrogen, Karlsruhe, Germany). The mononuclear cell fraction was incubated with the beads for 10 minutes at 4°C. Bead-bound cells were isolated by eight times washing with Dulbecco's PBS (PAA) in a magnetic field. After that, the beads were removed from the isolated T lymphocytes using Detachabeads (Invitrogen) at incubation for 45 minutes at 20°C. The purified cells were more than 99% CD3 positive and more than 97% positive for CD8, as detected by flow cytometry.

The isolated CD8^+ ^T lymphocytes were activated by anti-CD3 and anti-CD28 antibodies (both BD Pharmingen, Erembodegem, Belgium). Multi-well plates according to the kind of experiment were coated with 10 μg/ml of each of these antibodies in PBS at 4°C over night. Cells were seeded at a concentration of 8 × 10^5 ^cells/ml in RPMI with 10% FCS and 1% penicillin/streptomycin solution (all components were from PAA, Pasching, Austria). The cells were incubated for three days, without or with the daily addition of 0.1, 1, 10 or 100 ng/ml IL-2 (Invitrogen, Nivelles, Belgium). Maximum activation of the cells (as measured by the activation markers) was reached after four days, but the granularity (as measured by the sideward scatter) reached the maximum already after three days and decreased to day four.

In addition, the neurotransmitters norepinephrine (10 μM; Sigma-Aldrich, Taufkirchen, Germany), substance P (1 μM; Calbiochem, Bad Soden, Germany) and dopamine (1 μM; Sigma-Aldrich) were added each day. These were the lowest concentrations with maximum effect with regard to cell migration and activation (data not shown). The same concentrations were used in publications on CD8^+ ^T lymphocytes and natural killer cells [33], as well as neutrophil granulocytes and tumor cells [44-46]. The blood and plasma concentrations of the neurotransmitters used herein are usually reported to be 10 to 1,000 fold lower than those concentrations that we have used in our experiments, but local concentrations of the neurotransmitters can be much higher, e.g. the norepinephrine level was found to reach 500 μM at nerve terminals [47].

### Flow cytometry

Besides the above mentioned analysis of the purity of isolated CD8^+ ^T lymphocytes, flow cytometrical measurements were performed for the detection of the activation status of the cells using a FACSCalibur flow cytometer (Becton Dickinson, Heidelberg, Germany). The activation status of the cells was analysed by measuring the activation markers CD25 and CD45R0 with a fluorescein isothiocyanate (FITC)-coupled anti-human CD25 antibody and a phycoerythrin (PE)-coupled anti-human CD45R0 antibody (both Coulter Immunotech, Marseille, France). To determine changes of the CXCR1 expression during activation, cells were stained with a mouse anti-human CXCR1 or CXCR2 antibody (R&D Systems, Wiesbaden, Germany), and detected with Cy5-conjugated Fab fragment goat anti-mouse IgG (Dianova, Hamburg, Germany). In addition, the part of dead and viable cells was measured by propidium iodide staining.

### Immunoblotting

For the investigation of transcription factor activation, immunoblotting with phospho-specific antibodies was performed. Samples of 3 × 10^5 ^cells were incubated in 96-well plates coated with anti CD3- and anti CD28 antibodies or mouse IgG1 isotype control as described above. After the indicated time points the plates were spun down at 1200 rpm for 2 min. The supernatant was removed and cell lysis was performed using 30 μl of Laemmli buffer without β-mercaptoethanol. Lysates were incubated at 95°C 10 min prior to application to gel electrophoresis according to Laemmli [48]. The proteins were transferred to an Immobilion-P membrane (Millipore, Bedford, Mass.) [49], and immunoblotting was performed as described previously [44]. Phospho(Thr202/Tyr204)-Erk1/2, Erk1/2, phospho(Ser536)-p65, p65, phospho(Ser133)-CREB, CREB, Lamin A/C, phospho(Ser32)-IκBα, IκBα, and β-actin antibodies were obtained from New England Biolabs (Frankfurt a. M., Germany) and used at a concentration of 1:1,000 for Erk1/2, CREB and Lamin A/C, 1:500 for phospho-p65, p65, phospho-IκBα and Iκ Bα, and 1:2,000 for β-actin. Primary antibodies were detected using horseradish peroxidase-conjugated secondary anti-mouse or anti-rabbit antibodies (Southern Biotech, Birmingham, AL) at a concentration of 1:10,000. The luminescence signal was induced with chemiluminescence blotting substrate (Roche, Mannheim, Germany) and detected using a Hamamatsu C4742-98 system (Hamamatsu, Herrsching, Germany). Staining signals were quantified using the ImageJ software (NIH, Bethesda, MD). After detection of phospho-proteins membranes were stripped and reprobed with the corresponding non-phospho-antibody or antibodies against β-actin and Lamin A/C as standards.

For isolation of the nuclear fraction, hypotonic lysis was performed for 15 min on ice in 10 mM HEPES, 1.5 mM MgCl_2_, 1.5 mM NaF, 1 mM Na_3_VO_4_, 1 mM PMSF, protease inhibitor cocktail (Sigma-Aldrich), and 0.5% Triton X-100. Nuclei were collected by centrifugation at 14,000 g at 4°C and resuspended in Laemmli buffer without β-mercaptoethanol.

For the analysis of neurotransmitter receptors on naïve or activated CD8^+ ^T cells we used the following antibodies from Santa Cruz Biotechnology (Santa Cruz, California) at a dilution of 1:500: Goat anti NK-1R (clone N-19), goat anti D1DR (clone C-20), rabbit anti D2DR (clone H-50), rabbit anti D3DR (clone H-50), goat anti D4DR (clone N-20), goat anti D5DR (clone C-20), rabbit anti β1-adrenergic receptor (clone V-19), rabbit anti β1-adrenergic receptor (clone H-20).

### Semi-quantitative RT-PCR

Cells were activated in anti-CD3- and anti-CD28-coated wells with or without neurotransmitters for 24 h. Total RNA from 5 × 10^6 ^cells per sample was isolated using the Macherey Nagel total RNA isolation kit XS (Düren, Germany). An amount of 1.5 μg RNA was used for first-strand cDNA synthesis (Fermentas, St. Leon-Rot, Germany). cDNA samples were fivefold diluted serially (1:125; 1:625; 1:3,125; 1:15,625) and analyzed by semi-quantitative reverse transcriptase PCR using the following primers (5'→3'): IL-2, Fwd-ACTCACCAGGATGCTCACAT and Rev-AGGTAATCCATCTGTTCAGA; β-actin, Fwd-GTGGGGCGCCCCAGGCACCA and Rev-CTCCTTAATGTCACGCACGATTTC [50]. Primers were chosen to ensure the template spanning at least one intron so that any genomic DNA contamination would result in a larger product band. PCR products were visualized on a 1% ethidium bromide agarose gel.

### Migration

We performed our conventional three-dimensional, collagen-based migration assay as described in detail previously [51]. In brief, a suspension of 2 × 10^5 ^CD8^+ ^T lymphocytes in 50 μl RPMI with or without neurotransmitters was mixed with 100 μl of a buffered collagen solution (pH 7.4), containing 1.67 mg/ml bovine collagen type I (Invitrogen, Cohesion Technologies, Palo Alto, CA). The suspension was filled into self constructed migration chambers, which consist of a microscopic glass slide, wax walls, and a cover slip on top. After polymerization of the collagen at 37°C in a humidified 5% CO_2 _atmosphere, the migration of the cells was recorded by time-lapse videomicroscopy for 1 hour at 37°C. The paths of 30 randomly selected cells were digitized by computer-assisted cell tracking and the part of migratory active cells was calculated for each one minute interval [51].

### Flow-through adhesion assay

We have recently developed a new adhesion assay under flow conditions on the basis of the above described migration assay [52]. Human endothelial cells from the pulmonary microvasculature (HMVEC; Lonza, Verviers, Belgium) were used for the generation of a vascular structure. The cells were cultured up to six passages in EBM-2 medium with supplements (Lonza) in a humidified atmosphere at 5% CO_2_. For the experiments the HMVECs were seeded on collagen IV-coated flow chambers (μ-chamber I, IBIDI, Munich, Germany) in normal culture medium and incubated to confluency for two days. Before each experiment, the endothelium was activated by treatment with 10 ng/ml IL-1β (Invitrogen, Nivelles, Belgium) for 4 hours. The activated T lymphocytes (3 × 10^5 ^cells/ml in endothelial basal medium (PAA) with 2% fetal calf serum) were drawn through the flow chamber by a perfusion pump (Perfusor IV, B. Braun Melsungen AG, Melsungen, Germany) at a flow rate of 12.2 ml/h, which results in a shear stress of 0.25 dyne/cm^2 ^and represents physiological blood flow conditions in small vessels. The investigated neurotransmitters were added directly to the experiments at 10 μM for norepinephrine and 1 μM for dopamine and substance P. In blocking experiments, the IL-8 receptors CXCR1 and CXCR2 on CD8^+ ^T lymphocytes were blocked with mouse anti-human antibodies provided by R&D Systems at a concentration of 5 μg/ml each for 15 min prior to an experiment, IgG2a mouse-antibody served as isotype control. The suspension flow was digitally recorded by a video-camera mounted on the microscope and a connected computer. The number of adhesive cells and of rolling cells was analyzed. The adhesive cells were counted as the absolute number during the entire recording period of 20 minutes. The rolling cells were calculated per minute.

### Cytokine ELISA

To estimate the amount of secreted IL-2 2 × 10^6 ^CD8^+ ^T lymphocytes were activated for two days in 250 μl culture medium with or without neurotransmitters.

The amount of IL-8 secreted by HMVEC was estimated after stimulation of cell monolayers with 10 μM norepinephrine, 1 μM dopamine or 1 μM substance P alone or in combination with 10 ng/ml IL-1β (Invitrogen) for 4 hours in media without supplements.

Supernatant was collected and the concentration of IL-2 and IL-8 was measured by enzyme-linked immunoassays using the Quantakine kits (R&D Systems) according to the manufacturers protocol.

### Degranulation measured by β-hexosamidase release

The β-hexosamidase assay was performed as described by Shen *et al. *[53] with some modifications. Briefly, CD8^+ ^T lymphocytes were plated in a 96-well plate (3.5 × 10^5 ^cells/well), which was coated with an anti-CD3 or IgG2a antibody, and cultured in 100 μl RPMI (w/o FCS and PhenolRed) containing the neurotransmitter at above mentioned concentration. After 4 hours, the cells were harvested by centrifugation and 50 μl of the supernatant were mixed with 150 μl of a 1 mM para-nytrophenyl N-acetyl-β-D glucosamide (Sigma-Aldrich) solution in 0.1 M citrate buffer (pH 4.5). After 2 hours of incubation at 37°C the reaction was stopped by addition of 100 μl of 1 M Na_2_CO_3_. Absorbance was read at 405 nm using a Model 550 microplate reader (Bio-Rad Laboratories, Hercules, CA). Maximum release (positive control) was induced by cell lysis with 1% Triton X-100. β-Hexosamidase release was calculated as the percentage of total enzyme activity in Triton X-100-treated samples.

### Statistics

Significant changes were calculated using the Student's *t *test (two-tailed, unpaired). A probability value of p < 0.05 was accepted as statistically significant throughout the experiments.

## Authors' contributions

CS performed most of the experiments. AS and PB established the method of T cells activation. KL participated in performing the experiments. BN supported the evaluation of data with regard to cell migration and adhesion to endothelium under flow-conditions. KSZ participated in conceiving and coordinating the study. FE conceived the study and wrote the manuscript together with CS. All authors read and approved the final manuscript.

## References

[B1] Felten DL Direct innervation of lymphoid organs: substrate for neurotransmitter signaling of cells of the immune system Neuropsychobiol 1993 28 110 2 10.1159/000119011 7902965

[B2] Fink T Weihe E Multiple neuropeptides in nerves supplying mammalian lymph nodes: messenger candidates for sensory and autonomic neuroimmunomodulation? Neurosci Lett 1988 90 39 44 10.1016/0304-3940(88)90783-5 2457855

[B3] Lang K Bastian P Neurotransmitter effects on tumor cells and leukocytes Prog Exp Tumor Res 2007 39 99 121 full_text 17314504 10.1159/000100070

[B4] Sloan EK Capitanio JP Cole SW Stress-induced remodeling of lymphoid innervation Brain Behav Immun 2008 22 15 21 10.1016/j.bbi.2007.06.011 17697764 PMC2754291

[B5] Kin NW Sanders VM It takes nerve to tell T and B cells what to do J Leukoc Biol 2006 79 1093 104 10.1189/jlb.1105625 16531560

[B6] Lucin KM Sanders VM Jones TB Malarkey WB Popovich PG Impaired antibody synthesis after spinal cord injury is level dependent and is due to sympathetic nervous system dysregulation Exp Neurol 2007 207 75 84 10.1016/j.expneurol.2007.05.019 17597612 PMC2023967

[B7] Kohm AP Sanders VM Norepinephrine and beta 2-adrenergic receptor stimulation regulate CD4+ T and B lymphocyte function in vitro and in vivo Pharmacol Rev 2001 53 487 525 11734616

[B8] Grebe KM Hickman HD Irvine KR Takeda K Bennink JR Yewdell JW Sympathetic nervous system control of anti-influenza CD8+ T cell responses Proc Natl Acad Sci USA 2009 106 5300 5 10.1073/pnas.0808851106 19286971 PMC2664017

[B9] Weihe E Nohr D Michel S Muller S Zentel HJ Fink T Krekel J Molecular anatomy of the neuro-immune connection Int J Neurosci 1991 59 1 23 10.3109/00207459108985446 1774130

[B10] Barili P Bronzetti E Felici L Ferrante F Ricci A Zaccheo D Amenta F Age-dependent changes in the expression of dopamine receptor subtypes in human peripheral blood lymphocytes J Neuroimmunol 1996 71 45 50 10.1016/S0165-5728(96)00127-0 8982102

[B11] Levite M Chowers Y Ganor Y Besser M Hershkovits R Cahalon L Dopamine interacts directly with its D3 and D2 receptors on normal human T cells, and activates beta1 integrin function Eur J Immunol 2001 31 3504 12 10.1002/1521-4141(200112)31:12<3504::AID-IMMU3504>3.0.CO;2-F 11745370

[B12] Watanabe Y Nakayama T Nagakubo D Hieshima K Jin Z Katou F Hashimoto K Yoshie O Dopamine selectively induces migration and homing of naive CD8+ T cells via dopamine receptor D3 J Immunol 2006 176 848 56 16393968 10.4049/jimmunol.176.2.848

[B13] Nakano K Higashi T Hashimoto K Takagi R Tanaka Y Matsushita S Antagonizing dopamine D1-like receptor inhibits Th17 cell differentiation: preventive and therapeutic effects on experimental autoimmune encephalomyelitis Biochem Biophys Res Commun 2008 373 286 91 10.1016/j.bbrc.2008.06.012 18558081

[B14] Hasko G Szabo C Nemeth ZH Deitch EA Dopamine suppresses IL-12 p40 production by lipopolysaccharide-stimulated macrophages via a beta-adrenoceptor-mediated mechanism J Neuroimmunol 2002 122 34 9 10.1016/S0165-5728(01)00459-3 11777541

[B15] Saha B Mondal AC Majumder J Basu S Dasgupta PS Physiological concentrations of dopamine inhibit the proliferation and cytotoxicity of human CD4+ and CD8+ T cells in vitro: a receptor-mediated mechanism Neuroimmunomodulation 2001 9 23 33 10.1159/000049004 11435749

[B16] Torres KC Antonelli LR Souza AL Teixeira MM Dutra WO Gollob KJ Norepinephrine, dopamine and dexamethasone modulate discrete leukocyte subpopulations and cytokine profiles from human PBMC J Neuroimmunol 2005 166 144 57 10.1016/j.jneuroim.2005.06.006 16026859

[B17] Ghosh MC Mondal AC Basu S Banerjee S Majumder J Bhattacharya D Dasgupta PS Dopamine inhibits cytokine release and expression of tyrosine kinases, Lck and Fyn in activated T cells Int Immunopharmacol 2003 3 1019 26 10.1016/S1567-5769(03)00100-0 12810359

[B18] Herpfer I Lieb K Substance P receptor antagonists in psychiatry: rationale for development and therapeutic potential CNS Drugs 2005 19 275 93 10.2165/00023210-200519040-00001 15813642

[B19] Tuncer LI Alacam T Oral B Substance P expression is elevated in inflamed human periradicular tissue J Endod 2004 30 329 32 10.1097/00004770-200405000-00006 15107644

[B20] Kraneveld AD Nijkamp FP Tachykinins and neuro-immune interactions in asthma Int Immunopharmacol 2001 1 1629 50 10.1016/S1567-5769(01)00099-6 11562057

[B21] Felten DL Felten SY Bellinger DL Lorton D Noradrenergic and peptidergic innervation of secondary lymphoid organs: role in experimental rheumatoid arthritis Eur J Clin Invest 1992 22 Suppl 1 37 41 1281104

[B22] Levite M Nerve-driven immunity. The direct effects of neurotransmitters on T- cell function Ann N Y Acad Sci 2000 917 307 21 11268358 10.1111/j.1749-6632.2000.tb05397.x

[B23] Bost KL Pascual DW Substance P: a late-acting B lymphocyte differentiation cofactor Am J Physiol 1992 262 C537 45 1372476 10.1152/ajpcell.1992.262.3.C537

[B24] Jain J Loh C Rao A Transcriptional regulation of the IL-2 gene Curr Opin Immunol 1995 7 333 42 10.1016/0952-7915(95)80107-3 7546397

[B25] Karin M Liu Z Zandi E AP-1 function and regulation Curr Opin Cell Biol 1997 9 240 6 10.1016/S0955-0674(97)80068-3 9069263

[B26] Koike T Yamagishi H Hatanaka Y Fukushima A Chang JW Xia Y Fields M Chandler P Iwashima M A novel ERK-dependent signaling process that regulates interleukin-2 expression in a late phase of T cell activation J Biol Chem 2003 278 15685 92 10.1074/jbc.M210829200 12595531

[B27] Tran K Merika M Thanos D Distinct functional properties of IkappaB alpha and IkappaB beta Mol Cell Biol 1997 17 5386 99 9271416 10.1128/mcb.17.9.5386 PMC232389

[B28] Gergely L Cook L Agnello V A simplified method for Ca2+ flux measurement on isolated human B cells that uses flow cytometry Clin Diagn Lab Immunol 1997 4 70 4 9008284 10.1128/cdli.4.1.70-74.1997 PMC170478

[B29] Franklin RA Tordai A Patel H Gardner AM Johnson GL Gelfand EW Ligation of the T cell receptor complex results in activation of the Ras/Raf-1/MEK/MAPK cascade in human T lymphocytes J Clin Invest 1994 93 2134 40 10.1172/JCI117209 8182145 PMC294346

[B30] Romashkova JA Makarov SS NF-kappaB is a target of AKT in anti-apoptotic PDGF signalling Nature 1999 401 86 90 10.1038/43474 10485711

[B31] Feske S Calcium signalling in lymphocyte activation and disease Nat Rev Immunol 2007 7 690 702 10.1038/nri2152 17703229

[B32] Macian F NFAT proteins: key regulators of T-cell development and function Nat Rev Immunol 2005 5 472 84 10.1038/nri1632 15928679

[B33] Lang K Drell TL Niggemann B Zanker KS Entschladen F Neurotransmitters regulate the migration and cytotoxicity in natural killer cells Immunol Lett 2003 90 165 172 10.1016/j.imlet.2003.09.004 14687720

[B34] Bierer BE Burakoff SJ T cell adhesion molecules Faseb J 1988 2 2584 90 2838364 10.1096/fasebj.2.10.2838364

[B35] Hemler ME Jacobson JG Brenner MB Mann D Strominger JL VLA-1: a T cell surface antigen which defines a novel late stage of human T cell activation Eur J Immunol 1985 15 502 8 10.1002/eji.1830150515 2986987

[B36] Rot A Endothelial cell binding of NAP-1/IL-8: role in neutrophil emigration Immunol Today 1992 13 291 4 10.1016/0167-5699(92)90039-A 1510812

[B37] Tanaka Y Adams DH Shaw S Proteoglycans on endothelial cells present adhesion-inducing cytokines to leukocytes Immunol Today 1993 14 111 5 10.1016/0167-5699(93)90209-4 8466625

[B38] Cinamon G Grabovsky V Winter E Franitza S Feigelson S Shamri R Dwir O Alon R Novel chemokine functions in lymphocyte migration through vascular endothelium under shear flow J Leukoc Biol 2001 69 860 6 11404368

[B39] Tanaka Y Adams DH Hubscher S Hirano H Siebenlist U Shaw S T-cell adhesion induced by proteoglycan-immobilized cytokine MIP-1 beta Nature 1993 361 79 82 10.1038/361079a0 7678446

[B40] Takata H Tomiyama H Fujiwara M Kobayashi N Takiguchi M Cutting edge: expression of chemokine receptor CXCR1 on human effector CD8+ T cells J Immunol 2004 173 2231 5 15294933 10.4049/jimmunol.173.4.2231

[B41] Nishimura M Umehara H Nakayama T Yoneda O Hieshima K Kakizaki M Dohmae N Yoshie O Imai T Dual functions of fractalkine/CX3C ligand 1 in trafficking of perforin+/granzyme B+ cytotoxic effector lymphocytes that are defined by CX3CR1 expression J Immunol 2002 168 6173 80 12055230 10.4049/jimmunol.168.12.6173

[B42] Chuntharapai A Lee J Hebert CA Kim KJ Monoclonal antibodies detect different distribution patterns of IL-8 receptor A and IL-8 receptor B on human peripheral blood leukocytes J Immunol 1994 153 5682 8 7527448

[B43] Levite M Towards unveiling the mystery: from stressed brain/mind to outburst of disease Curr Opin Pharmacol 2008 8 458 9 10.1016/j.coph.2008.06.017 18644465

[B44] Bastian P Posch B Lang K Niggemann B Zaenker KS Hatt H Entschladen F Phosphatidylinositol 3-kinase in the G protein-coupled receptor-induced chemokinesis and chemotaxis of MDA-MB-468 breast carcinoma cells: a comparison with leukocytes Mol Cancer Res 2006 4 411 21 10.1158/1541-7786.MCR-06-0030 16778088

[B45] Lang K Drell TL Lindecke A Niggemann B Kaltschmidt C Zaenker KS Entschladen F Induction of a metastatogenic tumor cell type by neurotransmitters and its pharmacological inhibition by established drugs Int J Cancer 2004 112 231 8 10.1002/ijc.20410 15352035

[B46] Bastian P Balcarek A Altanis C Strell C Niggemann B Zaenker KS Entschladen F The inhibitory effect of norepinephrine on the migration of ES-2 ovarian carcinoma cells involves a Rap1-dependent pathway Cancer Lett 2009 274 218 24 10.1016/j.canlet.2008.09.008 18849110

[B47] Shimizu N Hori T Nakane H An interleukin-1 beta-induced noradrenaline release in the spleen is mediated by brain corticotropin-releasing factor: an in vivo microdialysis study in conscious rats Brain Behav Immun 1994 8 14 23 10.1006/brbi.1994.1002 8003768

[B48] Laemmli UK Cleavage of structural proteins during the assembly of the head of bacteriophage T4 Nature 1970 227 680 5 10.1038/227680a0 5432063

[B49] Towbin H Staehelin T Gordon J Electrophoretic transfer of proteins from polyacrylamide gels to nitrocellulose sheets: procedure and some applications Proc Natl Acad Sci USA 1979 76 4350 4 10.1073/pnas.76.9.4350 388439 PMC411572

[B50] Sakkas LI Scanzello C Johanson N Burkholder J Mitra A Salgame P Katsetos CD Platsoucas CD T cells and T-cell cytokine transcripts in the synovial membrane in patients with osteoarthritis Clin Diagn Lab Immunol 1998 5 430 7 9665944 10.1128/cdli.5.4.430-437.1998 PMC95595

[B51] Bastian P Lang K Niggemann B Zaenker KS Entschladen F Myosin regulation in the migration of tumor cells and leukocytes within a three-dimensional collagen matrix Cell Mol Life Sci 2005 62 65 76 10.1007/s00018-004-4391-6 15619008 PMC11924536

[B52] Strell C Lang K Niggemann B Zaenker KS Entschladen F Surface molecules regulating rolling and adhesion to endothelium of neutrophil granulocytes and MDA-MB-468 breast carcinoma cells and their interaction Cell Mol Life Sci 2007 64 3306 16 10.1007/s00018-007-7402-6 17994288 PMC11136373

[B53] Shen DT Ma JS Mather J Vukmanovic S Radoja S Activation of primary T lymphocytes results in lysosome development and polarized granule exocytosis in CD4+ and CD8+ subsets, whereas expression of lytic molecules confers cytotoxicity to CD8+ T cells J Leukoc Biol 2006 80 827 37 10.1189/jlb.0603298 16891618

